# *Clostridium butyricum* Ameliorates the Effect of Coprophagy Prevention on Hepatic Lipid Synthesis in Rabbits via the Gut–Liver Axis

**DOI:** 10.3390/ijms242417554

**Published:** 2023-12-16

**Authors:** Zhichao Li, Mengjuan Chen, Ran Zhang, Zhitong Wang, Hui He, Zhiyi Wan, Hengjian Li, Hanfang Cai, Zhi Chen, Ming Li, Huifen Xu

**Affiliations:** 1 College of Animal Science and Technology, Henan Agricultural University, Zhengzhou 450046, China; lizhichao@stu.henau.edu.cn (Z.L.); chen_333666979@163.com (M.C.); zr13237610911@163.com (R.Z.); 18963717463@163.com (Z.W.); h18375399868@163.com (H.H.); hegnjianli@126.com (H.L.); caihanfang.cool@163.com (H.C.); 2 College of Biological Sciences, China Agricultural University, No. 2 Yuan Ming Yuan West Road, Beijing 100193, China; gxmwzy@126.com; 3 College of Animal Science and Technology, Yangzhou University, Yangzhou 225000, China; zhichen@yzu.edu.cn

**Keywords:** coprophagy prevention, growth performance, hepatic lipid synthesis, *Clostridium butyricum*, gut–liver axis, rabbits

## Abstract

Coprophagy prevention (CP) affects the growth performance, hepatic lipid synthesis, and gut microbiota in rabbits. Supplementation with *Clostridium butyricum* (*C. butyricum,* Strain number: CCTCC M 2019962) has been found to improve growth performance in rabbits. However, it remains unknown whether *C. butyricum* can ameliorate the effects of CP on hepatic lipid synthesis and the underlying mechanisms are yet to be elucidated. Therefore, this study aimed to investigate the impact of CP on hepatic lipid synthesis and the underlying mechanism based on the gut–liver axis. The findings revealed that supplementation with *C. butyricum* could reverse CP-related growth performance, lipid accumulation, bile acid synthesis, and inflammation. Furthermore, *C. butyricum* exerted protective effects on the gut by preserving intestinal barrier integrity and modulating gut microbiota composition; these factors may represent potential mechanisms through which *C. butyricum* improves CP-related outcomes. Specifically, *C. butyricum* reshaped the microbiota by increasing butyric acid levels, thereby maintaining secondary bile acid (deoxycholic acid, chenodeoxycholic acid) balance and attenuating the inhibitory effects of the FXR/SHP pathway on lipid synthesis (SREBP1c/ApoA1). Moreover, the activation of butyrate/GPR43pathway by *C. butyricum* reduced damage to the intestinal barrier (ZO-1/Occludin/Claudin1) and restored the gut immune microenvironment in CP rabbits. In summary, supplementation with *C. butyricum* can alleviate the adverse effects of CP on growth performance and hepatic lipid synthesis by modulating the gut–liver axis.

## 1. Introduction

In rabbits, coprophagy is necessary to provide essential nutrients and affects the gut’s microbiota, energy metabolism, and cognitive behavior [1,2,3]. Some studies showed that coprophagy prevention (CP) reduces growth performance and hepatic lipid synthesis in New Zealand white rabbits [4,5]. Wang et al. further observed a correlation between the gut microbiota and lipid metabolism in New Zealand white rabbits [5]. However, the mechanism via which CP affects hepatic lipid metabolism by altering the intestinal microbiota remains unknown.

The liver plays a crucial role in lipid metabolism, and bile acids (BAs) are important for the digestion and absorption of lipids [6,7]. The gut–liver axis refers to the bidirectional relationship between the gut, the microbiota, and the liver [8,9]. The interaction between the gut and the liver is established through the portal vein, which carries gut-derived products to the liver. Moreover, BAs produced by the liver feed back to the gut via the biliary duct [8]. A proportion of BAs are then converted to secondary BAs by the gut microbiota, although most BAs are reabsorbed in the ileum and transported back to the liver via the portal circulation [10]. BAs activate the protein expression of farnesoid X receptor (FXR), which, in turn, leads to a reduction in liver and serum triglyceride (TG) levels [11,12]. This process occurs through the FXR-SHP-SREBP1c pathway [12].

In addition to lipid metabolism, BAs affect the composition of the gut microbiota [9,13]. The gut microbiota also plays an important role in maintaining the homeostasis of the gut–liver axis through microbiota-generated metabolites, such as short-chain fatty acids (SCFAs) and secondary BAs [9,13,14]. A recent study has shown that daily administration of acetate can reverse some of the effects caused by CP in Brandt’s vole [3]. 

Supplementation with *Clostridium butyricum* (*C. butyricum*), a butyric acid-producing bacterium, improves growth performance and the balance of the intestinal barrier in pigs and rabbits [15,16]. In the gut, *C. butyricum* produces secondary SCFAs and BAs [17,18,19]. Recently, Huang et al. conducted a study demonstrating that supplementation with *C. butyricum* maintains mucosal barrier integrity and improves growth performance in rabbits [16]. However, it is not clear how supplementation with *C. butyricum* affects rabbits treated with CP. Here, we hypothesized that supplementation with *C. butyricum* could rescue the reduction in growth performance and hepatic lipid synthesis caused by CP. We also focused on the changes in BAs and SCFAs in the gut–liver axis.

## 2. Results

### 2.1. Growth Performance and Meat Quality

**Figure 1:**
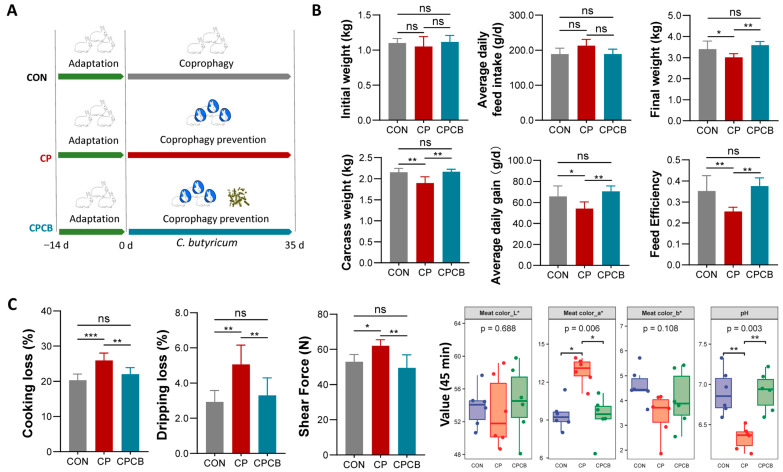
Effects of CP and CPCB on growth performance and meat quality. (**A**) Schematic of the experimental design. (**B**) The growth performance in different groups. (**C**) The meat quality (cooking loss, dripping loss, shear force, meat color brightness (L*), redness (a*), and yellowness (b*) values and pH at 45 min) in the different groups. CON: control; CP: coprophagy prevention; CPCB: CP + *C. butyricum*. Data are presented as the means ± standard deviations; *n* = 6. * *p* < 0.05, ** *p* < 0.01, and *** *p* < 0.001.

There was no difference in the initial weight of the groups (Figure 1B). Compared to the CON group, although CP did not affect the average daily feed intake, it significantly decreased the feed efficiency, average daily gain, final weight, and carcass weight (Figure 1B). CP also significantly affected muscle quality, as indicated by the elevated muscle cooking loss, dripping loss, shear force, meat color redness, and decreased pH at 45 min (Figure 1C). However, supplementation with *C. butyricum* resulted in a significant improvement in these characteristics caused by CP (Figure 1B,C).

### 2.2. Serum Biochemistry Analysis

**Figure 2:**
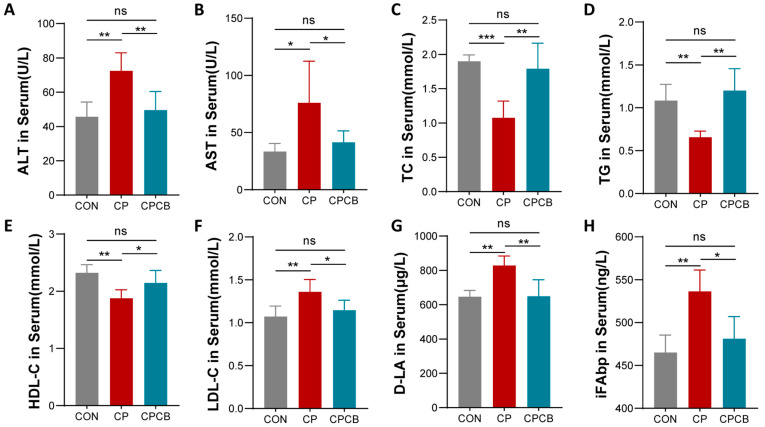
Effects of CP and CPCB on serum biochemistry. (**A**–**H**) Serum concentrations of ALT, AST, TC, TG, HDL-C, LDL-C, D-LA, and iFABP in the different groups. CON: control; CP: coprophagy prevention; CPCB: CP + *C. butyricum*. Data are presented as the means ± standard deviations; *n* = 6. * *p* < 0.05, ** *p* < 0.01, and *** *p* < 0.001.

Compared to the CON group, the CP group exhibited significantly increased concentrations of ALT, AST, LDL-C, D-LA, and iFABP in the serum and significantly reduced concentrations of TC, TG, and HDL-C in the serum (Figure 2A–H). However, these parameters were significantly restored in the CPCB group (Figure 2A–H).

### 2.3. Metabolite Analysis in the Cecal Contents

**Figure 3:**
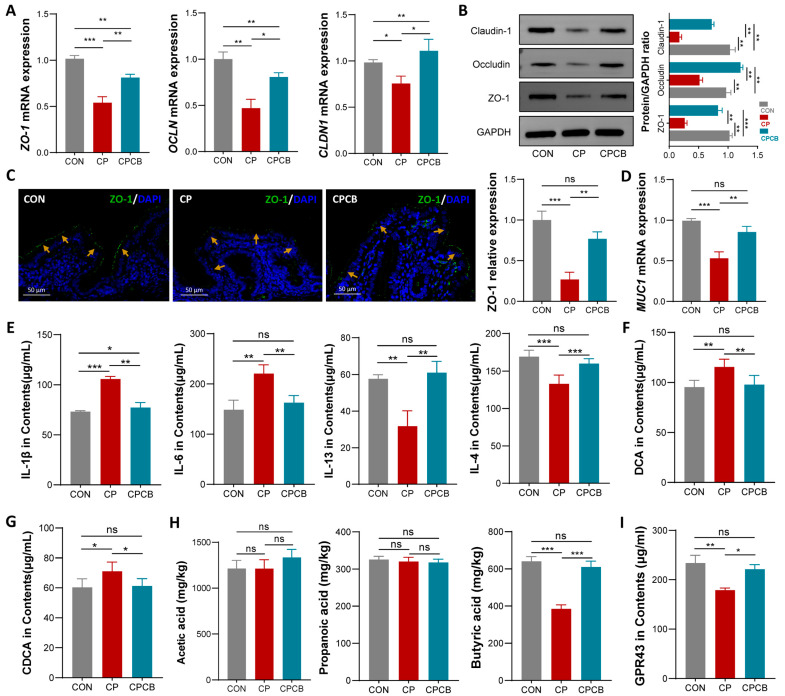
Effects of CP and CPCB on metabolites in the cecal contents. (**A**) The mRNA levels of tight junction proteins (*ZO-1*, *OCLN*, and *CLDN1*) in the cecum. (**B**) The protein levels of tight junction proteins (ZO-1, OCLN, and CLDN1) in the cecum. (**C**) Immunofluorescence staining analysis of ZO-1 (green) protein expression in the cecum; blue fluorescence represents the nucleus (Bar, 50 μm), yellow arrows: ring-like structure of ZO-1 in the cecum. (**D**) The mRNA level of *MUC 1* in the cecum. (**E**–**I**) The concentrations of IL-1β, IL-6, IL-13, IL-4, deoxycholic acid (DCA), chenodeoxycholic acid (CDCA), acetic acid, propanoic acid, butyric acid, and GPR43 in the cecal contents. CON: control; CP: coprophagy prevention; CPCB: CP + *C. butyricum*. Data are presented as the means ± standard deviations; *n* = 6. * *p* < 0.05, ** *p* < 0.01, and *** *p* < 0.001.

Increased serum levels of D-LA and iFABP suggested an impaired cecal mucosal barrier. Therefore, we examined the cecal mucosal barrier proteins and immune-related factors in the cecal contents. Compared to the CON group, the mRNA and protein expression levels of genes related to the cecal mucosal barrier (*ZO-1*, *OCLN*, and *CLDN1*) were significantly decreased in the CP group (Figure 3A,B). The immunofluorescence staining revealed that the ring-like structure of ZO-1 in the cecum was disrupted and reduced in size in the CP group compared with the CON group (Figure 3C). The mRNA level of *MUC1* was also significantly decreased in the CP group compared to the CON group (Figure 3D). In the cecal contents, IL-1β and IL-6 were significantly increased in the CP group compared to the CON group (Figure 3E). However, the CP group exhibited significant decreases in the IL-13 and IL-4 levels compared to those in the CON group (Figure 3E). Notably, these indicators were all alleviated in the CPCB group compared to the CP group (Figure 3A–E). These results suggested that supplementation with *C. butyricum* can effectively alleviate CP-induced cecal mucosal barrier damage, reduce intestinal mucosal permeability, and improve the immune environment. In the cecal contents, the levels of deoxycholic acid (DCA) and chenodeoxycholic acid (CDCA) were significantly increased in the CP group compared to the CON group (Figure 3F,G), while the levels of butyric acid and GPR43 were significantly decreased in the CP group (Figure 3H,I). Notably, these indicators were also all alleviated in the CPCB group compared to the CP group (Figure 3F–I). The levels of acetic acid and propanoic acid in the cecal contents did not differ between the groups (Figure 3F).

### 2.4. Metabolite Analysis in Liver

**Figure 4:**
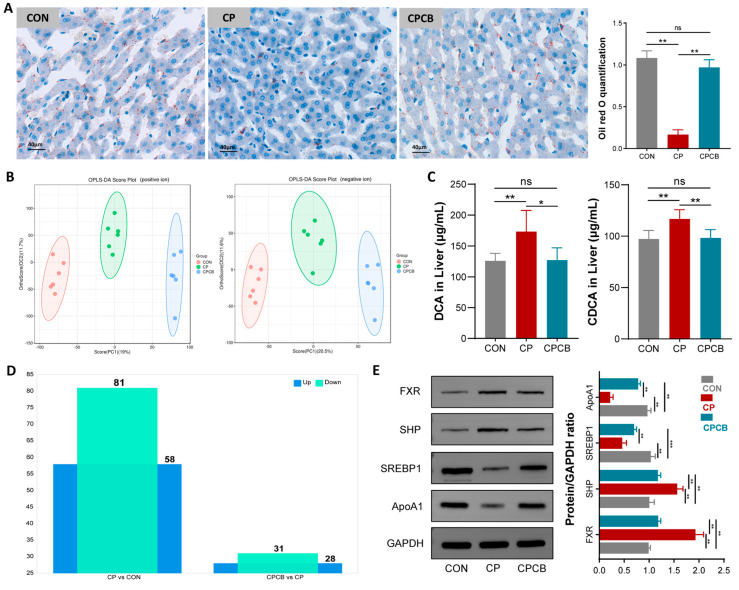
Metabolite analysis in the liver. (**A**) Concentrations of hepatic lipids in the different groups. Lipid droplets were stained with Oil red O and measured as a percentage of the area. (**B**) Orthogonal partial least squares−discriminant analysis (OPLS−DA) of metabolites. (**C**) Differentially accumulated metabolites between the groups. (**D**) The contents of deoxycholic acid (DCA) and chenodeoxycholic acid (CDCA) were detected via ELISA. (**E**) Protein expression levels of the FXR−SHP−SREBP1c pathway members in the liver. CON: control; CP: coprophagy prevention; CPCB: CP + *C. butyricum*. Data are presented as the means ± standard deviations; *n* = 6. * *p* < 0.05, ** *p* < 0.01, and *** *p* < 0.001.

Serum analyses indicated that lipid synthesis in the liver was impaired in the CP group. The increased intestinal permeability caused by CP can also lead to more BAs flowing back into the liver through the portal vein. Therefore, metabolite analysis was further performed in liver. Oil red O staining showed that lipid droplet accumulation in the liver was significantly lower in the CP group than in the CON group, indicating that CP affected lipid metabolism in the livers of rabbits (Figure 4A). However, the lipid droplets in the livers of the CPCB group were significantly higher than those of the CP group and comparable to those of the CON group (Figure 4A). OPLS-DA based on the nontargeted metabolomics assay showed that the metabolites were significantly different between the groups (Figure 4B). Of the 139 differentially accumulated metabolites (DAMs) identified between the CP and CON groups, 58 (41.73%) and 81 (58.27%) metabolites were upregulated and downregulated, respectively. Of the 59 DAMs identified between the CPCB and CP groups, 28 (47.46%) and 31 (52.54%) metabolites were upregulated and downregulated, respectively (Figure 4C). Among these DAMs, the concentrations of DCA and CDCA exhibited significant increases in the CP group compared to the CON group. In contrast, the concentrations of DCA and CDCA were significantly decreased in the CPCB group compared to the CP group (Table S1). We further quantified the levels of DCA and CDCA using ELISA, and the results were consistent with the findings of the LC-MS/MS analysis (Figure 4D).

To elucidate the underlying mechanisms via which CP affects hepatic lipid metabolism, we examined the gene expression levels of the FXR-SHP-SREBP1c pathway components in liver. Western blot analysis showed the higher protein expression of SHP and FXR and lower protein expression of ApoA1 and SREBP1c in the CP group than in the CON group (Figure 4E). Notably, these changes were alleviated in the CPCB group compared to the CP group (Figure 4E).

### 2.5. Microbiome Analysis of the Cecal Contents

**Figure 5:**
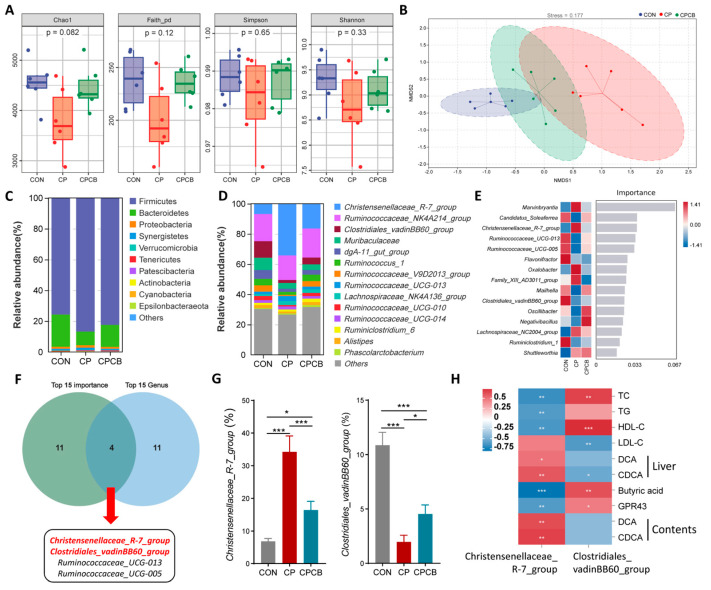
Effects of CP and CPCB on the microbial community in the cecal contents. (**A**) Alpha diversity analysis among the groups. (**B**) Nonmetric multidimensional scaling (NMDS) analysis among the groups (stress = 0.177). The relative abundance of bacterial phyla (**C**) and genera (**D**). (**E**) Top 15 marker species identified via random forest analysis. The abscissa represents the importance of the species to the classifier model. (**F**) Venn diagram detailing the top 15 species in terms of abundance and importance. (**G**) Relative abundances of *Christensenellaceae_R*−*7_group* and *Clostridiales_vadinBB60_group*. Data are presented as the means ± standard deviations; *n* = 6. (**H**) Correlation analysis between the cecal microbiota and metabolites. Red, positive correlation. Blue, negative correlation. CON: control; CP: coprophagy prevention; CPCB: CP + *C. butyricum*. * *p* < 0.05, ** *p* < 0.01, and *** *p* < 0.001.

As some primary BAs are converted to secondary BAs by the gut microbiota [10], we examined the composition of the gut microbiota using 16S RNA sequencing. Alpha diversity analysis showed no significant differences between the groups (Figure 5A). NMDS analysis showed a different microbial composition between the CON and CP groups. The CPCB group exhibited an intermixed microbial community between those of the CON and CP groups (Figure 5B). Compared to the CON group, the relative abundance of *Firmicutes* increased from 79.55% to 86.85% in the CP group, and the relative abundance of *Bacteroidetes* decreased from 17.32% to 8.78% in the CP group (Figure 5C). The top 15 genera with the highest abundances are shown in Figure 5D, and the top 15 genera with importance analyzed via the random forest model are shown in Figure 5E. Venn analysis of the top 15 species ranked by abundance and importance identified four species, namely *Christensenellaceae_R-7_group*, *Ruminococcaceae_UCG-013*, *Ruminococcaceae_UCG-005*, and *Clostridiales_vadinBB60_group* (Figure 5F). Compared to the CON group, the relative abundance of *Christensenellaceae_R-7_group* was significantly higher in the CP group. Furthermore, the relative abundance of *Clostridiales_vadinBB60_group* was significantly lower (Figure 5G). Notably, the relative abundances of *Christensenellaceae_R-7_group* and *Clostridiales_vadinBB60_group* were significantly decreased and increased, respectively, in the CPCB group compared to the CP group (Figure 5G). Correlation analysis further revealed that *Clostridiales_vadinBB60_group* was positively correlated with butyric acid and negatively correlated with DCA and CDCA, while *Christensenellaceae_R-7_group* was significantly negatively correlated with butyric acid and positively correlated with DCA and CDCA (Figure 5H).

## 3. Discussion

Our findings confirmed that CP decreased hepatic lipid synthesis and growth performance in New Zealand white rabbits, which is consistent with a previous report [4,5]. Dietary *C. butyricum* supplementation can alleviate CP-induced cecal barrier damage, reduce cecal mucosal permeability, restore hepatic lipid synthesis, and improve growth performance. In addition, a positive effect of *C. butyricum* supplementation on cooking loss, dripping loss, shear force, and pH at 45 min of meat was observed. The gut–liver axis may be involved in the underlying mechanism of this phenomenon.

The composition of gut microbes is critical to maintaining the homeostasis of the gut–liver axis. *C. butyricum* is a butyrate-producing bacterium that has been used as a feed additive for monogastric and aquatic animals [20,21,22]. It has also been used for decades as a probiotic bacterium [23]. It produces secondary BAs and SCFAs (such as butyric acid) in the gut [17,18,19]. DCA is a typical secondary BA converted from CA by the 7-α-dehydroxylase produced by *Clostridium* spp. in the gut [18,19]. SCFAs and BAs are pivotal molecules in the gut–liver axis and can indirectly or directly influence the physiological functions of the liver [13]. In this study, metagenomic sequencing analysis showed that the relative abundance of *Clostridiales_vadinBB60_group* was strikingly decreased in the CP group, while the *Christensenellaceae_R-7_group* abundance was significantly increased in the CP group. The content of butyric acid in the cecal contents was also significantly decreased in the CP group.

Butyrate induces the expression of tight junction (TJ) proteins that maintain intestinal mucosal barrier function [24,25]. Dietary *C. butyricum* supplementation increased the expression of TJ proteins in the cecal mucosa of rabbits [16]. In line with these findings, our results showed a notable decrease in the expression of TJ proteins in the cecum of the CP group. Compared to expression levels in the CP group, the expression levels of TJ proteins were increased in the CPCB group. MUC1 is widely expressed in the mucosal epithelium and is an important component of the mucosal barrier [26]. Consistent with the down-regulation of TJ protein expression, the mRNA level of *MUC1* was significantly reduced in the cecum of the CP group. The mucosal barrier acts as a site for the interactions between the gut and the liver, restraining the spread of microbes and metabolites [8]. Mucosal barrier dysfunction increases intestinal permeability, which may increase the hepatic reflux of BAs through the portal vein. In this study, metabolite analysis based on LC–MS/MS showed that the concentrations of DCA and CDCA in the liver were significantly increased in the CP group compared to the CON group, which was further validated by the results obtained via ELISA. Compared to the CP group, the concentrations of DCA and CDCA in the liver were significantly decreased in the CPCB group.

In the liver, BAs, such as CDCA, DCA, and CA, activate FXR and further induce the protein expression of the small heterodimer partner (SHP), thereby suppressing the protein expression of SREBP-1c and leading to a decrease in hepatic TG, plasma TG, and plasma lipoproteins [12,27,28,29]. Consistent with previous reports, our results showed that the protein expression levels of SHP and FXR were increased in the liver in the CP group compared to the CON group, while the protein expression levels of ApoA1 and SREBP1c were decreased in the CP group. Correlation analysis revealed that the abundance of *Clostridiales_vadinBB60_group* was positively correlated with the presence of butyric acid, plasma TG, plasma TC, and plasma HDL-C and negatively correlated with the presence of DCA and CDCA. Conversely, *Christensenellaceae_R-7_group* abundance had a negative effect on the levels of plasma TG, plasma TC, and plasma HDL-C. These findings are consistent with previous reports indicating that *Christensenellaceae* is negatively correlated with body mass index and serum lipid levels, including TG and TC [30,31]. Furthermore, as a SCFA-producing microbe, *Clostridiales_vadinBB60_group* is positively linked to the production of SCFAs [32].

## 4. Materials and Methods

### 4.1. Animal Ethics

All procedures used in this study were reviewed and approved by the Ministry of Science and Technology in China (2014). The Institutional Animal Care and Use Committee (IACUC) of Henan Agricultural University (permit number: 22-0203) was followed for all procedures relating to the handling, care, and management of live rabbits.

### 4.2. Animals and Samples

Male New Zealand white rabbits were obtained from Huaxing Experimental Animal Farm (Zhengzhou, Henan, China). As shown in Figure 1A, a total of 18 rabbits with a similar body weight (1.08 ± 0.1 kg, 4 weeks of age) were randomly divided into 3 groups of 6 rabbits each: the control (CON) group, the CP group, and the CP and *C. butyricum* addition (CPCB) group. The basic formulas and nutritional levels of the diets were defined as previously described [33]. In the CON group, rabbits were provided with a basal diet. In the CP and CPCB groups, rabbits wore a collar (7 cm) to prevent them from consuming soft feces. In the CPCB group, rabbits with collars were fed a basal diet supplemented with *C. butyricum* F06 (1 × 10^9^ colony-forming units (CFU) per kg). The *C. butyricum* F06 strain has been deposited in the China Center for Type Culture Collection and assigned the following deposition number: CCTCC M 2019962.

After a period of five weeks, the rabbits were euthanized through cervical dislocation. The liver, cecum, and cecal contents were collected; snap-frozen in liquid nitrogen; and stored at −80 °C for future analysis. Blood samples were collected using serum tubes without anticoagulants. Serum was subsequently obtained through centrifugation (3000× *g* for 15 min at 4 °C) and then stored at −80 °C. A portion of the liver sample was fixed in a 4% paraformaldehyde solution for histological examination.

### 4.3. Growth Performance Analysis

The full amount of the diet was weighed each day, and the remaining diet was weighed again the next day. At the end of the experiment, the total feed intake of each rabbit was calculated. The initial weight of the rabbits was measured at the beginning of the trial, and the final weight was obtained at the end of the trial. The feed efficiency was calculated as feed efficiency = average daily gain/average daily feed intake.

Rabbit hind leg muscles were used to evaluate meat quality. Muscle samples were cut parallel to the direction of the muscle fibers (4 cm × 2 cm × 1 cm) and accurately weighed for the cooking and dripping loss tests. For the cooking loss test, the samples were cooked in a water bath at 85 °C for 10 min (min); then, these samples were cooled to 25 °C, and the residual surface water was gently removed using filter paper. The weight of the samples was subsequently recorded. By quantifying the difference between the weights of the raw and heat-treated samples, cooking loss was determined and expressed as a percentage of the original sample weight. All samples were cooked in one batch, and the mean of three technical replicates was taken as the data for each sample. For the dripping loss test, the samples were suspended in a plastic box. After storage at 4 °C for 24 h, the samples were dried with filter paper and weighed again. Dripping loss was calculated as the difference between the weights before and after refrigeration, and it is expressed as a percentage of the initial weight. For the measurement of shear force, three strips measuring 1.2 cm × 1.0 cm × 8.0 cm were cut parallel to the muscle fibers for testing. The shear force was measured using a meat tenderness tester (RH-N50, Runhu, Guangzhou, China). The highest force was recorded as the shear force when cutting the rabbit leg samples.

The pH of the meat 45 min after slaughter was measured via the direct insertion of a pH meter electrode (Testo205, Testo, Lenzkirch, Germany). The pH meter was calibrated with pH 4.00 and 6.86 buffer at 25 °C. Meat samples were blanched for 45 min at 25 °C. Meat color at 45 min after slaughter was recorded using a colorimeter (Opto-Star, Matthaus, Nürnberg, Germany) according to the CIE LAB trichromatic system, namely L* (lightness), a* (redness), and b* (yellowness). The colorimeter was calibrated using an illuminant of D65, an observer angle of 10°, and an aperture size of 5.0 mm, and the average color was obtained by measuring the same muscle sample three times at three different locations.

### 4.4. Histological Analysis

Liver samples were fixed in 4% paraformaldehyde and embedded in optimal cutting temperature (OCT) compound. Then, cryosectioning was performed to produce sections of 10 µm in thickness. The cryosections were stained with Oil red O working solution for 10 min at room temperature. The cryosections were counterstained with hematoxylin for 3 min. Then, the cryosections were treated with a bluing reagent (G1040) for 1 s. These sections were observed under a light microscope (Nikon Eclipse Ti-SR, Tokyo, Japan) and evaluated using Image-Pro Plus 6.0 software (Media Cybernetics, Rockville, MD, USA).

### 4.5. Serum Biochemical Analyses

Serum biochemical parameters, including aspartate aminotransferase (AST), alanine aminotransferase (ALT), total cholesterol (TC), total triglyceride (TG), high-density lipoprotein cholesterol (HDL-C), and low-density lipoprotein cholesterol (LDL-C), were detected using a clinical chemistry analyzer (SD1, Seamaty, Chengdu, China).

### 4.6. Cytokine and Metabolite Assays

Enzyme-linked immunosorbent assay (ELISA) kits (Jiangsu Meimian Industrial Co., Ltd., Suzhou, China) were used to detect the contents of D-lactic acid (D-LA), intestinal fatty acid binding protein (iFABP), IL-1β, IL-13, IL-4, IL-6, and G protein-coupled receptor 43 (GPR43). Butyric acid, acetic acid, and propanoic acid in the cecal contents were detected via Agilent 7890A gas chromatography (Agilent, Santa Clara, CA, USA) at Bioyigene Biotechnology Co., Ltd. (Wuhan, China).

Metabolites in the liver were detected using liquid chromatography-tandem mass spectrometry (LC–MS/MS). Liver samples were slowly thawed at 4 °C and homogenized in 1 mL of precooled methyl alcohol/acetonitrile/water (2:2:1, *v*/*v*). After ultrasonic decomposition at 4 °C for 30 min, the samples were incubated for 10 min at −20 °C to precipitate the protein and then centrifuged (14,000× *g*, 4 °C, 20 min). The resulting supernatant was carefully collected and subjected to vacuum drying before being stored at −80 °C. The sample was redissolved in 100 μL of an acetonitrile/water mixture (1:1, *v*/*v*), adequately vortexed, and then centrifuged for 15 min (14,000× *g*, 4 °C). The resulting supernatant was separated using ultra-high-performance liquid chromatography (UHPLC, 1290 Infinity LC, Agilent, USA). Quality control (QC) samples were included in the analysis queue to evaluate the system stability and data reliability throughout the entire experimental procedure. Samples were detected in both positive and negative electrospray ionization (ESI) modes. Analyses were performed using a UHPLC system coupled with a TripleTOF 6600 mass spectrometer (AB Sciex, Foster City, CA, USA).

The raw MS data were transformed into MzXML files using ProteoWizard MS Convert. Subsequently, XCMS was utilized for feature detection, retention time correction, and alignment. The metabolites were determined using accurate mass (<25 ppm) and MS/MS data, which were matched with our standard database. Within the extracted ion features, only the variables exhibiting more than 50% of the nonzero measurement values in at least one group were retained for subsequent analysis. The software SIMCA-P 14.1 (Umetrics, Umea, Sweden) was used for orthogonal partial least squares discriminant analysis (OPLS-DA). The identification criteria for differentially accumulated metabolites (DAMs) were set as *p* < 0.05 and variable influence on projection (VIP) value > 1.

### 4.7. Quantitative Polymerase Chain Reaction (qPCR)

Total RNA extraction and qPCR were performed as previously described [34]. Each sample was performed with three technical replicates. The primer sequences for RT–PCR (Table 1) were designed using the Primer 6.0 software and synthesized by Sangon Biotech Co., Ltd. (Shanghai, China). PCR amplification efficiency was assessed using a standard curve. Data were shown as the fold-change in gene expression in each group versus the control.

### 4.8. Western Blot

Western blotting was performed as previously described [3]. The primary antibodies included ZO-1 (1:1000, #13663, CST, Danvers, MA, USA), Occludin (1:1000, #91131, CST, Danvers, MA, USA), Claudin (1:1000, #13255, CST, Danvers, MA, USA), FXR (1:500, GCP41, Bioworld, Bloomington, MN, USA), SHP (1:500, BS6827, Bioworld, Bloomington, MN, USA), SREBP1 (1:500, BS66105, Bioworld, Bloomington, MN, USA), ApoA1 (1:1000, GB112563, Servicebio, Wuhan, Hubei, China), and GAPDH (1:1000, #2118, CST, Danvers, MA, USA). The membranes were then treated with secondary antibodies, including HRP-linked anti-mouse IgG (1:2000, #7076, CST, Danvers, MA, USA) and HRP-linked anti-rabbit IgG (1:2000, #7074, CST, Danvers, MA, USA), for 2 h at room temperature. The blots were visualized using a LAS4000 chemiluminescence system (Fujifilm, Tokyo, Japan). The ImageJ 1.8 software was used to analyze the densities. The data are presented as the fold-change in protein expression in each group versus the control.

### 4.9. Immunofluorescence Analysis

Sections (5-millimeter-thick) of formalin-fixed paraffin-embedded tissue were obtained from the cecum sample. The sections were dewaxed, rehydrated, and underwent antigen retrieval. Bovine serum albumin was used for blocking the sections for 30 min at room temperature, followed by incubation overnight at 4 °C with primary antibody against ZO-1 (1:300, GB111402, Servicebio, Wuhan, China). The secondary antibody was then incubated for 1 h at room temperature. Nuclear counterstaining was performed using 4′,6-Diamidino-2-Phenylindole (DAPI) for 10 min. The sections were then observed under a fluorescence microscope. The images were evaluated using the Image-pro plus 6.0 software (Media Cybernetics, Rockville, MD, USA).

### 4.10. 16S rRNA Gene Sequencing

Genomic DNA extraction and 16S rRNA gene sequencing were performed as previously described [33]. In brief, the V3-V4 region of the 16S rRNA gene was amplified using the forward primer (5′-ACTCCTACGGGAGGCAGCA-3′) and the reverse primer (5′-GGACTACHVGGGTWTCTAAT-3′). Paired-end 250-base pair sequencing was performed via an Illumina NovaSeq 6000 platform (Illumina, San Diego, CA, USA) by Shanghai Personal Biotechnology Co., Ltd. (Shanghai, China). Sequencing reads were analyzed using Gene Cloud tools (https://www.genescloud.cn, 9 August 2022), which are based on QIIME2 software (v. 2021.11) [35]. Nonmetric multidimensional scaling (NMDS) analysis was performed based on the Bray–Curtis distance. Correlation analysis was carried out based on the Pearson correlation coefficient.

### 4.11. Statistical Analysis

Statistical analyses were performed using SPSS 22.0 software (SPSS Inc., Chicago, IL, USA). Due to the normal distribution of the data, one-way ANOVAs, followed by Tukey’s multiple comparison tests, were used for comparisons. Data were presented as the means ± standard deviations (SDs). * *p* < 0.05, ** *p* < 0.01, and *** *p* < 0.001.

## 5. Conclusions

**Figure 6:**
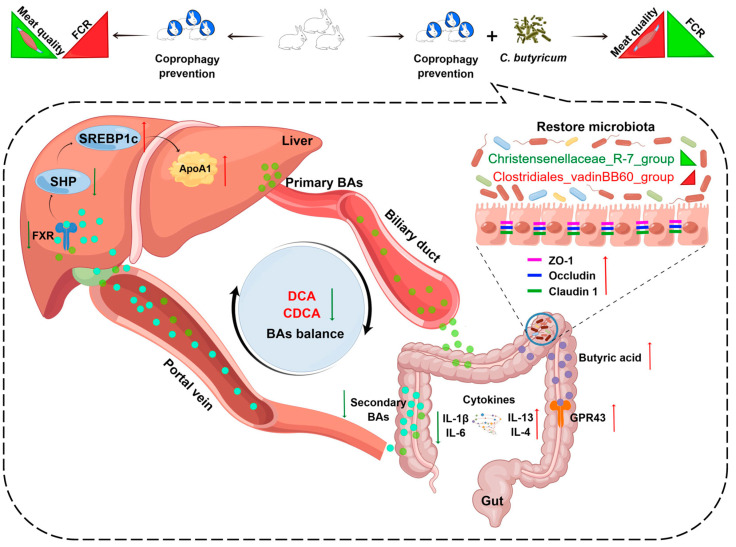
Diagram showing the mechanism via which supplementation with *C. butyricum* can alleviate the adverse effects of coprophagy prevention (CP) by modulating the gut–liver axis. CP results in an aberrant composition of the microbiome in the cecum. This leads to a reduction in the level of butyric acid and dysfunction in the cecal mucosal barrier, which increases intestinal permeability. Consequently, this condition facilitates an augmented influx of deoxycholic acid (DCA) and chenodeoxycholic acid (CDCA) through the portal system, which further inhibits hepatic lipid synthesis through FXR signaling. Red arrows: up-regulated, green arrows: down-regulated.

In conclusion, we found that CP leads to an abnormal cecal microbiome composition that causes decreased butyric acid in the cecal contents, cecal mucosal barrier dysfunction, increased intestinal permeability, and increased portal influx of DCA and CDCA (Figure 6). Elevated DCA and CDCA levels in the liver further inhibited hepatic lipid synthesis via FXR signaling (Figure 6). This study shows that supplementation with *C. butyricum* can alleviate the adverse effects of CP on the growth performance and hepatic lipid synthesis by modulating the gut–liver axis in rabbits.

## Figures and Tables

**Table 1:** Paired primers for qPCR.

Gene ID	Name	Primer Sequence (5′-3′)	bp	Amplification Efficiency (%)	R^2^ (%)
XM_017344772.1	*OCLN*	F: TCCGACTTCGTGGAGAGAGT R: TACTGCTGCTGCTCAAACGA	181	102.5	99.7
XM_017348359.1	*ZO-1*	F: TCCATAGAGACCGGCGTCA R: GGTTTTAGGATCACAGTGTGGC	222	103.2	99.1
NM_001089316.1	*CLDN1*	F: AGATGCGGATGGCTGTCAT R: AAGTAGGGCACCTCCCAGAA	203	107.5	98.6
XM_051858582.1	*MUC1*	F: TTCGGCACTGATTTCACAGA R: CAGAGGAGGGAGACAGAACATC	227	105.4	99.3
NM_001082253	*GAPDH*	F: CGATGCCCCCATGTTTGTGA R: TCATGAGCCCCTCCACAATG	149	106.8	99.6

## Data Availability

The 16S rRNA gene sequencing data in this study can be found in the Sequence Read Archive under project number PRJNA1007274.

## Supplementary Materials

The following supporting information can be downloaded via this link: https://www.mdpi.com/article/10.3390/ijms242417554/s1.

## Abbreviations

*C. butyricum*, *Clostridium butyricum*; CP, coprophagy prevention; CPCB, CP and C. butyricum addition; DCA, deoxycholic acid; CDCA, chenodeoxycholic acid; SHP small heterodimer partner; FXR, farnesoid X receptor; SREBP1c, sterol response element-binding protein-1c; ApoA1, apolipoprotein A-1; BAs, Bile acids; SCFAs, short-chain fatty acids; CFU, colony-forming units; AST, aspartate aminotransferase; FCR, feed conversion ratio; ALT, alanine aminotransferase; TC, total cholesterol; TG, total glyceride; TJ, tight junction; LDL-C, low-density lipoprotein cholesterol; HDL-C, high-density lipoprotein cholesterol; D-LA, D-Lactic acid; iFABP, intestinal fatty acid binding protein; IL-6, interleukin-6; IL-4, interleukin-4; IL-13, interleukin-13; IL-1β, interleukin-1β; GPR43, G protein-coupled receptor 43; LC-MS/MS, liquid chromatography-tandem mass spectrometry; DAMs, differentially accumulated metabolites; VIP, variable influence on projection.
